# Naturally Elevated Fe and Mn Degrade Groundwater Quality in Changfa Town, Hailun City, Songnen Plain: A Preliminary Hydrogeochemical and Health Risk Assessment

**DOI:** 10.3390/toxics14060495

**Published:** 2026-06-06

**Authors:** Zhiwei Yang, Ke Yang, Junbo Yu, Yangyang Chen, Kaiming Wang, Shaozhong Qiao, Jiayu Wang, Xinyi Wang, Jiacheng Liu, Xue Liu, Chenchen Wang

**Affiliations:** 1 Observation and Research Station of Earth Critical Zone in Black Soil, Ministry of Natural Resources, Harbin 150086, China; yangzhiwei@mail.cgs.gov.cn (Z.Y.);; 2 Harbin Natural Resources Survey, China Geological Survey, Harbin 150086, China; 3 School of Earth Sciences and Resources, China University of Geosciences, Beijing 100083, China; 4 Institute of Geophysical and Geochemical Exploration, Chinese Academy of Geological Sciences, Langfang 065000, China

**Keywords:** hydrochemistry, health risks, water quality index, rural area, Songnen Plain

## Abstract

Groundwater serves as a vital source of domestic and agricultural water in rural areas of the Songnen Plain. Its chemical composition and water quality directly impact public health and regional sustainable development, making them subjects of significant concern. This study employed a comprehensive analytical framework, integrating Piper trilinear diagrams, ionic ratio analysis, the Water Quality Index (WQI), and the Human Health Risk Assessment (HHRA) model, to preliminarily evaluate groundwater conditions in a rural township of the Songnen Plain. The multi-method approach was designed to provide scientific insights for groundwater pollution prevention and remediation strategies in the region. Results indicate that the predominant groundwater chemical type in the study area is HCO_3_-Ca. The hydrochemical process is primarily controlled by weathering and dissolution of silicate and carbonate minerals, accompanied by cation exchange. The WQI ranged from 84.78 to 192.82, with an average of 132.68, indicating overall moderate water quality. Fe and Mn are significant factors affecting water quality. The potential non-carcinogenic risks posed by groundwater to children, females, and males (0.988, 0.701, 0.534) and carcinogenic risks (1.77 × 10^−5^, 6.27 × 10^−5^, 4.81 × 10^−5^) are both below the USEPA recommended threshold (1.0, 1 × 10^−4^), indicating that the health risks were generally acceptable, though the HI for children approached the threshold. The results underscore the need for targeted mitigation of elevated Fe/Mn concentration (e.g., via aeration biofilters) while highlighting the region’s low health risks under current conditions. This work provides a template for integrating geochemical and health risk paradigms in groundwater management.

## 1. Introduction

Groundwater, as the largest accessible freshwater resource globally, plays a critical role in meeting human water demands and maintaining ecosystem health [1,2]. It is also a vital source of drinking water, providing approximately half of the world’s potable water supply, particularly in rural areas [3,4]. In China, over 70% of the rural population relies on untreated groundwater from private or communal wells as their primary source of daily water [5]. However, in recent years, intensive human activities have led to declining groundwater levels, reduced water availability, and deteriorating water quality [6,7], posing significant threats to water safety and public health in rural areas. Therefore, understanding the hydrochemistry and controlling factors of groundwater, as well as scientifically evaluating its quality, is essential for promoting effective water resource protection and sustainable utilization.

The Songnen Plain is known for its fertile soils [8] and serves as a critical grain production base in China [9]. The region depends heavily on groundwater for crop irrigation, which is essential for sustaining food production [10]. Additionally, groundwater serves as the main drinking water source for rural populations in the region, thus being essential for public health and safety. In recent years, extensive research has been conducted on groundwater in the Songnen Plain. The hydrochemistry of groundwater is primarily influenced by water-rock interactions and human activities [11], and identifying the controlling factors is of great significance for preventing water pollution [12]. Tian [13], using correlation analysis and Piper diagrams, identified that the hydrochemistry of groundwater in the Songnen Plain are primarily controlled by rock weathering and dissolution, with some influence from human activities. Bian [14], through ion ratio methods, found that the groundwater chemistry in the eastern and central irrigation region of the Songnen Plain is predominantly influenced by the weathering and dissolution of carbonates and sulfates, accompanied by cation exchange processes. Groundwater quality assessment is not only an essential tool for identifying water contamination characteristics but also serves as a foundation for human health risk evaluation. The Water Quality Index (WQI) is commonly applied for evaluating regional water quality [15]. Zhang [16] applied the WQI method and found that while groundwater across much of the Songnen Plain meets drinking water standards, localized areas remain unfit for consumption. To further explore the health impacts of groundwater consumption, human health risk assessment (HHRA) models have been introduced to quantify potential risks posed by different chemical components to human health [17,18,19]. Sun [20] analyzed nitrate-related health risks in groundwater from the Suihua region of the Songnen Plain, revealing that infants are at the highest risk, requiring specific attention. At present, as China’s township economies advance towards an eco-friendly circular development model, their requirements for water quality are correspondingly increasing. Therefore, conducting research on groundwater chemical characteristics and water quality assessment in townships across the Songnen Plain holds significant importance for supporting China’s rural revitalization and safeguarding national food security.

This study focuses on a typical township in the Songnen Plain to investigate the hydrochemistry and controlling factors of groundwater. A combination of Gibbs diagrams, Piper trilinear plots, and ionic ratio analysis was employed to characterize the hydrochemical composition of the groundwater. The WQI was employed to evaluate groundwater quality, and the health risks associated with key indicators were further assessed. These results are intended to inform groundwater protection strategies and support the pursuit of ecological sustainability in the rural communities of the study area.

## 2. Materials and Methods

### 2.1. Study Region

The study area, with a total area of 103 km^2^, lies in the northeastern Songnen Plain. The region experiences a mid-temperate continental monsoon climate, with an average annual temperature of approximately 2 °C. The annual precipitation ranges from 500 to 600 mm, with 88% of the rainfall occurring between May and September.

The stratigraphy in the study area, ordered from oldest to youngest, includes Late Cretaceous Nenjiang Formation, the Middle Pleistocene Shanghuangshan Formation, the Upper Pleistocene Guxiangtun Formation, the Holocene High Floodplain Deposits, and the Holocene Low Floodplain Deposits.

Groundwater in the study area is stored in three aquifer systems. From top to bottom, these are: (1) the Quaternary unconsolidated rock pore aquifer, (2) the Cretaceous clastic rock pore-fracture aquifer, and (3) the Lower metamorphic rock and granite fissure aquifer. The water samples collected in this study originate from the Cretaceous clastic rock pore-fracture aquifer, which consists of siltstone interbedded with thin gravel layers and is buried at depths ranging from 100 to 230 m. Groundwater in the aquifer is mainly recharged by precipitation and surface water, and discharges primarily through subsurface runoff and artificial extraction, following a regional flow direction from northeast to southwest [21].

### 2.2. Sampling and Analysis

**Figure 1:**
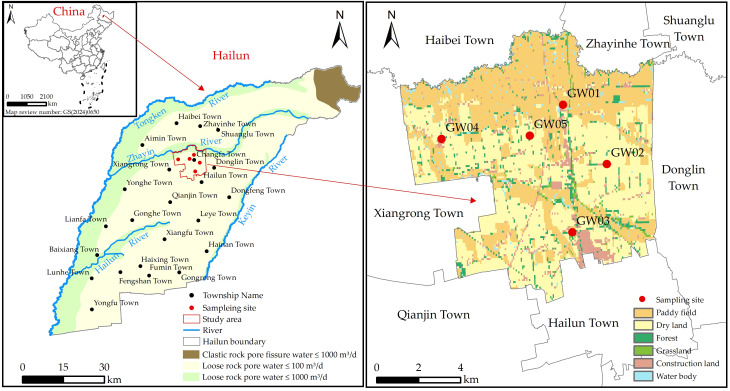
Map showing the location of Changfa Town within Hailun City and the Songnen Plain, and the spatial distribution of groundwater sampling sites. Well depths and aquifer information are provided in Table 1.

Five groundwater samples were collected from rural community supply wells in August 2024 (Figure 1; Table 1). At each site, well depth, elevation, groundwater type, and aquifer type were documented. Before collection, each well was purged for 15 min to obtain representative water. Sample containers were triple-rinsed with well water, and the collected water was passed through a 0.45-μm membrane filter and sealed immediately. All samples were kept at 4 °C and delivered to the laboratory within 24 h for analysis.

During sample collection, pH and total dissolved solids (TDS) were measured on-site using the HANNA HI129 EC/pH meter. All other test indicators were analyzed by the Harbin Natural Resources Survey. The tested parameters included total hardness (TH), Na^+^, K^+^, Mg^2+^, Ca^2+^, HCO_3_^−^, Cl^−^, SO_4_^2−^, F^−^, NH_4_^+^, NO_2_^−^, NO_3_^−^, As, Ba, Cr, Cu, Fe, Mn, Ni, Se, Zn, CN^−^, and volatile phenols. Analytical methods and their detection limits are listed in Table 2.

All chemicals used were of analytical grade or higher purity, and ultrapure water was employed throughout the analytical procedures. A systematic quality assurance/quality control protocol was implemented spanning field sampling, laboratory analysis, and data validation.

During field sampling, two blank samples were collected per batch to monitor potential contamination during sampling, transportation, and storage. All blank results were either below the method detection limits or at negligible levels, confirming the absence of significant contamination.

In the laboratory, analytical accuracy was verified using certified reference materials for all parameters, with relative errors within the acceptable ranges of the certified values. Precision was assessed through duplicate analysis of one sample per batch, with relative deviations meeting the quality control criteria; additionally, each sample was analyzed in duplicate, with the relative error between duplicates maintained within 5%. For each analytical batch, one procedural blank and one calibration standard were analyzed to monitor background interference and calibration accuracy, and the relative standard deviation of replicate analyses was maintained below 10%.

As a final validation step, the ionic balance error was calculated for each sample. The charge balance errors for the five groundwater samples were 0.78%, 1.66%, −1.10%, 0.75%, and 2.79%, confirming the overall reliability and acceptability of the analytical results.

### 2.3. WQI

The weight assigned to each parameter in the WQI calculation was determined based on its relative importance to drinking water quality, following the approach established by Pesce and Wunderlin [22] and subsequently adopted in groundwater quality studies in northeastern China by Tian et al. [21], Xiao et al. [23,24], and Gao et al. [25]. The specific calculation formula is as follows:

(1) $$W_{i}=\frac{w_{i}}{\sum_{i=1}^{n}w_{i}}$$

The relative weight of chemical element *W_i_* is defined as follows: *n* represents the total number of chemical elements considered in the evaluation, and *w_i_* denotes the weight assigned to chemical element *i* based on its significance to water quality and human health. The weights (*w_i_*) and relative weights (*W_i_*) of other chemical elements are presented in Table 3.

(2) $$q_{i}=\frac{C_{i}}{S_{i}}\times 100$$

where *q_i_* is the quality coefficient; *C_i_* represents the actual detected concentration of the chemical element in water (mg·L^−1^); and *S_i_* denotes the permissible limit for the chemical element in drinking water according to the Chinese National Standard for Drinking Water Quality (SDWQ) (mg·L^−1^) [26] and World Health Organization (WHO) (mg·L^−1^) [27].

(3) $$SI_{i}=W_{i}\times q_{i}$$

where *SI_i_* represents the mass index of chemical element *i*; *W_i_* denotes the relative weight of chemical element *i*; and *q_i_* is the mass coefficient of chemical element *i*.

(4) $$WQI=\sum_{i=1}^{n}SI_{i}$$

where *WQI* represents the water quality index, and *SI_i_* denotes the quality index of chemical element *i*. Based on the *WQI* values, water quality can be classified into five categories: when *WQI* < 50, the water quality is excellent; when 50 ≤ *WQI* < 100, the water quality is good; when 100 ≤ *WQI* < 200, the water quality is moderate; when 200 ≤ *WQI* < 300, the water quality is poor; and when *WQI* ≥ 300, the water quality is very poor and unsuitable for drinking or irrigation purposes [25]. The specific weight values are presented in Table 3. K^+^ was excluded from the WQI calculation because neither the SDWQ [26] nor the WHO [27] guidelines provide a permissible limit for potassium in drinking water and its concentration in the study area was consistently low, posing negligible influence on overall water quality.

**Table 3:** Weight and relative weight of the chemical elements.

Elements	Weight ($w_{i}$)	Relative Weight ($W_{i}$)	Units	Limit Values (*S_i_*)
pH	4	0.045	-	6.5–8.5
TDS	4	0.045	mg·L^−1^	1000
TH	2	0.023	mg·L^−1^	450
Na^+^	4	0.045	mg·L^−1^	200
K^+^	-	-	mg·L^−1^	-
Mg^2+^	3	0.034	mg·L^−1^	30 ^a^
Ca^2+^	3	0.034	mg·L^−1^	200 ^a^
HCO_3_^−^	2	0.023	mg·L^−1^	120 ^a^
Cl^−^	3	0.034	mg·L^−1^	250
SO_4_^2−^	3	0.034	mg·L^−1^	250
F^−^	4	0.045	mg·L^−1^	1.0
NH_4_^+^	5	0.057	mg·L^−1^	0.64 ^b^
NO_2_^−^	5	0.057	mg·L^−1^	3.0 ^a^
NO_3_^−^	5	0.057	mg·L^−1^	20
As	5	0.057	mg·L^−1^	0.01
Ba	2	0.023	mg·L^−1^	0.7
Cr	5	0.057	mg·L^−1^	0.05
Cu	3	0.034	mg·L^−1^	1.0
Fe	4	0.045	mg·L^−1^	0.3
Mn	4	0.045	mg·L^−1^	0.1
Ni	5	0.057	mg·L^−1^	0.02
Se	2	0.023	mg·L^−1^	0.01
Zn	3	0.034	mg·L^−1^	1.0
CN^−^	4	0.045	mg·L^−1^	0.05
Volatile Phenols	4	0.045	mg·L^−1^	0.002
SUM	88	1	-	-

^a^ HCO_3_^−^, Ca^2+^, Mg^2+^ and NO_2_^−^ guidelines are from the WHO [27]; ^b^, The NH_4_^+^ limit is converted from the NH_3_-N limit (0.5 mg·L^−1^) specified in SDWQ [26], using the conversion factor 18/14.

### 2.4. HHRA

Groundwater serves as the primary source of water for local residents, supporting both domestic and agricultural needs. However, the consumption of or dermal contact with contaminated groundwater may pose significant health risks to humans [28]. In this study, the human health risks associated with groundwater use were assessed using the HHRA model recommended by the United States Environmental Protection Agency (USEPA).

The exposure doses for ingestion and dermal contact pathways were calculated using the following equations [20]:

(5) $$ADD_{iing}=\frac{C_{i}\times IR\times ABS\times EF\times ED}{BW\times AT}$$

(6) $$ADD_{iderm}=\frac{C_{i}\times SA\times K_{p}\times EV\times ET\times EF\times ED\times CF}{BW\times AT}$$

(7) $$ADD=ADD_{iing}+ADD_{iderm}$$

where $ADD_{iing}$ is the average daily exposure dose of element *i* through the drinking pathway (mg·kg^−1^·d^−1^); $ADD_{iderm}$ is the average daily exposure dose of element *i* through dermal contact (mg·kg^−1^·d^−1^); ADD represents the total average daily exposure dose of element *i* (mg·kg^−1^·d^−1^); $C_{i}$ is the measured concentration of element *i*; other exposure dose parameters and their definitions are provided in Table 4.

The non-carcinogenic hazard index (HI) is a comprehensive evaluation of all non-carcinogenic elements present in the water samples. The calculation formula is as follows:

(8) $$HI=\sum_{i=1}^{n}HQ_{i}=\sum_{i=1}^{n}\frac{ADD_{i}}{RfD_{i}}$$

The total non-carcinogenic risk is represented by *HI*, while *HQ_i_* represents the non-carcinogenic risk of element *i*, and *RfD_i_* refers to the non-carcinogenic reference dose for element *i* (mg·kg^−1^·d^−1^), with values provided in Table 4. When *HI* ≤ 1.0, the non-carcinogenic risk can be considered negligible; however, when *HI* > 1.0, the non-carcinogenic risk cannot be ignored [31].

The carcinogenic risk index (*TCR*) provides a comprehensive assessment of all carcinogenic elements present in water samples. The calculation formula is as follows:

(9) $$TCR=\sum_{i=1}^{n}CR_{i}=\sum_{i=1}^{n}ADD_{i}\times SF_{i}$$

The total carcinogenic risk is represented as *TCR*; *CR_i_* represents the carcinogenic risk of element *i*; and *SF_i_* is the carcinogenic slope factor for element *i* (mg·kg^−1^·d^−1^), with values provided in Table 5. When *TCR* ≤ 1 × 10^−6^, it indicates no significant carcinogenic risk; when 1 × 10^−6^ < *TCR* ≤ 1 × 10^−4^, the carcinogenic risk is considered acceptable; and when *TCR* > 1 × 10^−4^, it indicates a significant carcinogenic risk [31].

## 3. Results

### 3.1. Hydrochemical Characteristics of Groundwater

The analytical results are presented in Table 6. NO_3_^−^, Cr, Cu, Ni, Se, CN^−^, and volatile phenols were below the method detection limits in all samples.

The statistical summary of hydrochemical parameters for all groundwater samples in the study area is presented in Table 7. The pH ranged from 6.96 to 7.31, with an average value of 7.11, indicating that the groundwater is slightly alkaline. According to the classification of TH, groundwater can be categorized as soft water (TH < 75 mg·L^−1^), moderately hard water (75 mg·L^−1^ ≤ TH < 150 mg·L^−1^), hard water (150 mg·L^−1^ ≤ TH < 300 mg·L^−1^), and very hard water (TH ≥ 300 mg·L^−1^) [36]. The TH ranged from 78.10 to 146.10 mg·L^−1^, with an average value of 103.88 mg·L^−1^, which classified it as moderately hard water. The TDS ranged from 162.80 to 366.00 mg·L^−1^, with an average value of 283.96 mg·L^−1^, indicating that the groundwater in the study area is generally freshwater (TDS < 1000 mg·L^−1^). The pH, TH, and TDS values were all below the limits set by the SDWQ and WHO.

The concentrations of major anions and cations in groundwater exhibited noticeable differences. As shown in Table 7, the average concentrations of major anions followed the order: HCO_3_^−^ > Cl^−^ > SO_4_^2−^, while the average concentrations of major cations were ranked as: Ca^2+^ > Na^+^ > Mg^2+^.

Fe, Mn, and As exceeded the SDWQ limits, with mean concentrations of 3.99, 0.96, and 0.0063 mg·L^−1^, respectively. In contrast, Ba and Zn remained well below their respective limits, averaging 0.18 and 0.020 mg·L^−1^.

Fluoride at low levels is beneficial for dental health but may cause fluorosis at elevated concentrations [37]. In this study, F^−^ ranged from 0.30 to 0.60 mg·L^−1^ (mean = 0.43 mg·L^−1^), remaining below the SDWQ limit of 1.0 mg·L^−1^.

NO_2_^−^ varied from ND to 0.038 mg·L^−1^ (mean = 0.012 mg·L^−1^), well below the WHO guideline of 3.0 mg·L^−1^. Nevertheless, its high coefficient of variation (1.28) points to considerable spatial variability, potentially reflecting localized anthropogenic inputs [38].

The NH_4_^+^ concentration ranged from 0.38 to 0.72 mg/L, with a mean of 0.47 mg·L^−1^. While the mean concentration was below the limit of 0.64 mg·L^−1^ stipulated in SDWQ, site GW05 (0.72 mg/L) exceeded this threshold, suggesting localized anthropogenic influence.

### 3.2. Groundwater Hydrochemical Types

**Figure 2:**
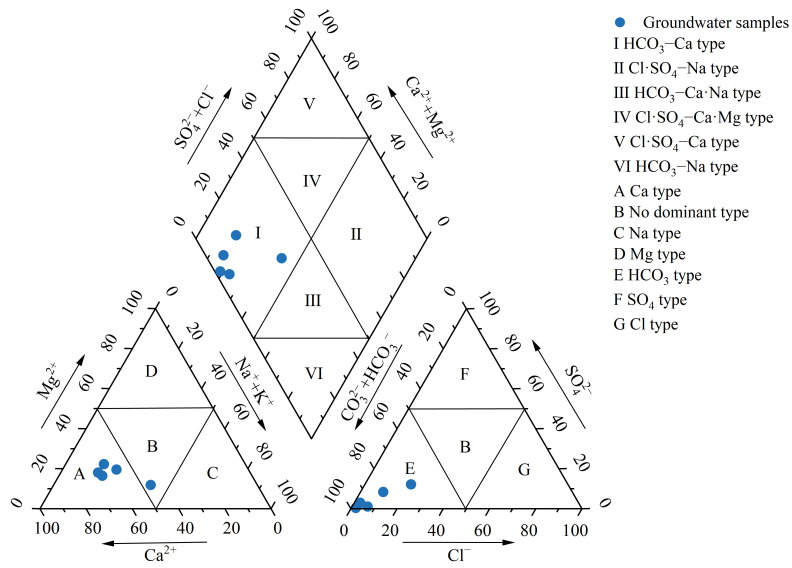
Piper trilinear diagram of groundwater samples from the study area, showing the dominant HCO_3_-Ca hydrochemical type. All five samples plot within the calcium-bicarbonate facies.

The Piper trilinear diagram provides a visual representation of the relative abundances of major ions and the resulting hydrochemical facies [39,40]. Figure 2 shows that Ca^2+^ and HCO_3_^−^ were the dominant cation and anion, respectively, defining the groundwater as predominantly HCO_3_-Ca type.

### 3.3. Groundwater Quality

Parameters below detection limits were assigned values equal to half their respective detection limits for the WQI computation. The resulting WQI values ranged from 84.78 to 192.82 (mean = 132.68), corresponding to overall moderate water quality: one sample fell in the “good” category, and the remaining four were classified as “moderate.” No sample reached the “excellent,” “poor,” or “very poor” categories.

### 3.4. Human Health Risk

Based on the HHRA model, non-carcinogenic and carcinogenic risks were evaluated for children, females, and males through two exposure pathways: drinking water intake and dermal contact.

**Figure 3:**
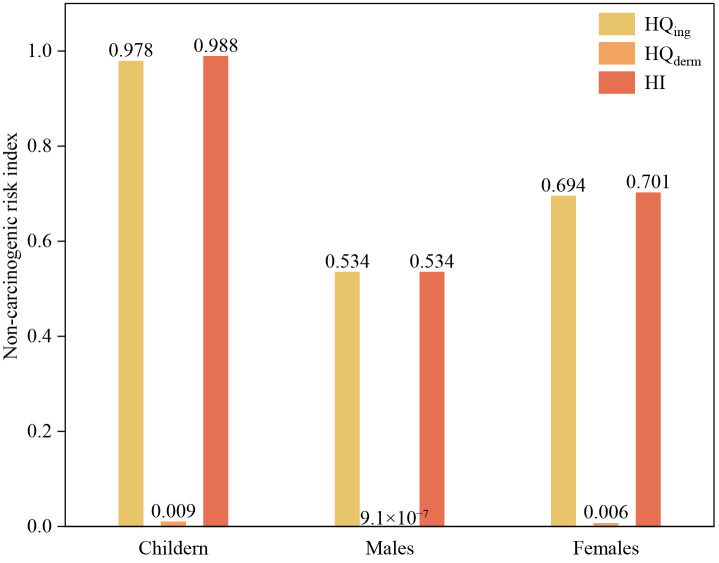
Hazard index (HI) values for different population groups, with stacked bars showing the contributions of the drinking water ingestion and dermal contact pathways.

As shown in Figure 3, the non-carcinogenic risks via the drinking water pathway were higher than that of the dermal contact pathway. In both pathways, the non-carcinogenic risk followed the order: children > females > males. Non-carcinogenic health risks across substances followed a consistent ranking for children, females, and males via drinking water and dermal exposure pathways: As > F^−^ > Mn > Fe > NO_2_^−^ > Zn.

As shown in Figure 3, the hazard index (HI) values decreased in the order of children (0.988) > females (0.701) > males (0.534), indicating that children were subject to the highest non-carcinogenic risk. Nevertheless, all HI values were below the USEPA threshold of 1.0, suggesting that the non-carcinogenic risks were acceptable.

The carcinogenic risk assessment (Table 8) revealed arsenic (As) as the dominant carcinogenic substance, with the risk via the drinking water pathway exceeding that via dermal contact. TCR followed the order: females > males > children, with all values below the acceptable threshold of 1.0 × 10^−4^, indicating that the carcinogenic risks were within acceptable limits.

## 4. Discussion

### 4.1. Analysis of Factors Controlling Groundwater Hydrochemistry

**Figure 4:**
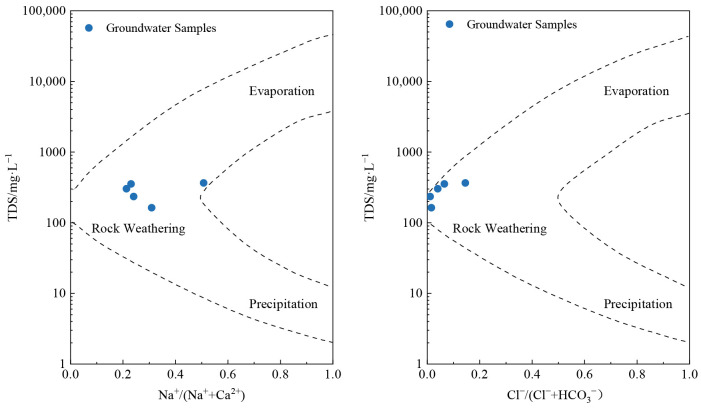
Gibbs diagram plotting Na^+^/(Na^+^ + Ca^2+^) and Cl^−^/(Cl^−^ + HCO_3_^−^) ratios against TDS. All groundwater samples fall within the rock weathering dominance zone, indicating that mineral weathering is the primary mechanism controlling groundwater chemistry. Blue dots are groundwater samples. Dashed lines partition three genetic zones: precipitation zone (below bottom dash), rock weathering zone (inside left dash), and evaporation zone (inside the upper double dashed envelope).

The Gibbs diagram, which plots the ratio of Na^+^/(Na^+^ + Ca^2+^) and Cl^−^/(Cl^−^ + HCO_3_^−^) against TDS, is widely used to identify the dominant mechanisms controlling groundwater chemistry: rock weathering, evaporation-crystallization, and atmospheric precipitation [41,42]. As shown in Figure 4, the groundwater samples fell within the rock weathering dominance zone, with TDS concentrations ranging from 100 to 1000 mg·L^−1^ and Na^+^/(Na^+^ + Ca^2+^) and Cl^−^/(Cl^−^ + HCO_3_^−^) ratios below 0.5. This indicates that mineral weathering is the primary process governing groundwater chemistry in the study area. Notably, TDS exhibited minimal variation as the Na^+^/(Na^+^ + Ca^2+^) and Cl^−^/(Cl^−^ + HCO_3_^−^) ratios increased, suggesting the occurrence of cation exchange processes. All samples were collected from the confined Cretaceous Nenjiang Formation aquifer; thus, the chemical composition of the groundwater was primarily determined by the weathering and dissolution of the siltstone and thin gravel layers within this formation. The confined nature and considerable burial depth (114–183 m) of the aquifer effectively minimized the influence of atmospheric precipitation and evaporation on groundwater chemistry, which is consistent with findings from other studies in the Songnen Plain [14,21].

**Figure 5:**
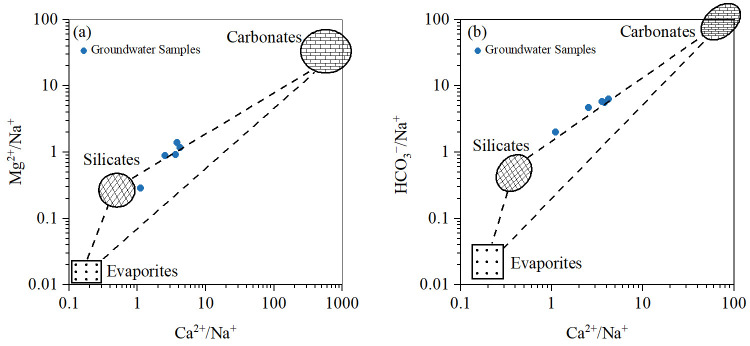
End-member mixing diagrams for discriminating silicate and carbonate weathering signatures: (**a**) Ca^2+^/Na^+^ vs. Mg^2+^/Na^+^; (**b**) Ca^2+^/Na^+^ vs. HCO_3_^−^/Na^+^ (ratios in meq/L). Groundwater samples plot between the silicate and carbonate end-members, indicating joint influence of both weathering processes. The dashed lines represent theoretical mixing lines connecting three characteristic end-members: silicates (left), carbonates (upper right), and evaporites (lower left).

Ion ratio analysis is widely used in water chemistry to trace water–rock interaction processes and identify the sources of dissolved ions in groundwater [43]. Having established that rock weathering is the dominant mechanism controlling groundwater chemistry, the milliequivalent ratios of Ca^2+^/Na^+^ versus Mg^2+^/Na^+^ and HCO_3_^−^/Na^+^ were employed to further discriminate between silicate and carbonate weathering end-members [44,45,46]. As shown in Figure 5, the groundwater samples plotted between the silicate and carbonate end-members, indicating that the groundwater chemistry was jointly influenced by the weathering and dissolution of silicate minerals (e.g., feldspar) and carbonate minerals (e.g., calcite). This interpretation is consistent with the lithology of the aquifer, which consists of siltstone interbedded with conglomerate.

**Figure 6:**
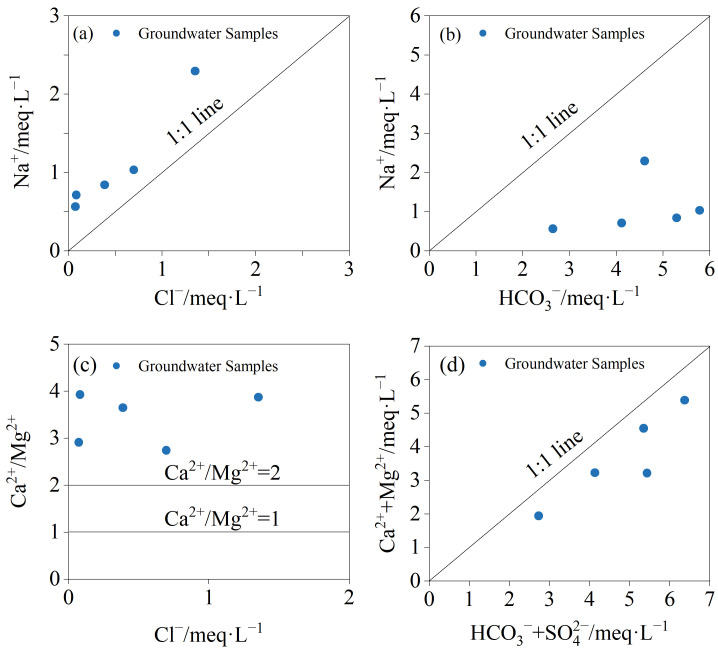
Ion ratio plots for tracing water–rock interaction processes (ratios in meq/L): (**a**) Na^+^ vs. Cl^−^ with 1:1 equivalence line; (**b**) Na^+^ vs. HCO_3_^−^ with 1:1 equivalence line; (**c**) Ca^2+^/Mg^2+^ vs. Cl^−^ with equivalent ratio lines; (**d**) (Ca^2+^ + Mg^2+^) vs. (HCO_3_^−^ + SO_4_^2−^) with 1:1 equivalence line. Blue dots represent groundwater samples.

The Na^+^/Cl^−^ equivalent ratio is widely used to infer the origin of groundwater and the sources of Na^+^ and Cl^−^ [47]. Marine-influenced atmospheric precipitation exhibits a Na^+^/Cl^−^ ratio of approximately 0.86, reflecting the marine aerosol signature. However, the natural range of Na^+^/Cl^−^ values in groundwater is relatively broad and depends on specific hydrogeological and geochemical conditions [48]. Importantly, the Na^+^/Cl^−^ ratio also serves as an indicator of the direction and intensity of ion-exchange processes. In waters occurring within active groundwater exchange zones, the Na^+^/Cl^−^ ratio may exceed 0.86, reflecting excess Na^+^ released through direct cation exchange, Ca^2+^ in groundwater replacing Na^+^ on the aquifer matrix, whereas Na^+^/Cl^−^ ratios below 0.86 are characteristic of residual brines or evaporative concentration that enriches Cl^−^ relative to Na^+^ [49]. A ratio above 0.86 generally indicates additional Na^+^ contributions from silicate weathering or direct cation exchange [47,50]. As shown in Figure 6a, all groundwater samples in the study area plotted well above the 1:1 equivalence line, with Na^+^/Cl^−^ ratios ranging from 1.49 to 8.72, confirming that Na^+^ was derived not only from halite dissolution but predominantly from the weathering of silicate minerals (e.g., albite) and cation exchange processes that release excess Na^+^. In the Na^+^/HCO_3_^−^ diagram (Figure 6b), all samples plotted below the 1:1 equivalence line, indicating that HCO_3_^−^ in the groundwater originated not only from silicate weathering but also from carbonate mineral dissolution, which is consistent with the findings shown in Figure 5.

Ca^2+^ and Mg^2+^ in the groundwater primarily originated from the weathering and dissolution of carbonate and silicate minerals [50,51]. All sampling points exhibited Ca^2+^/Mg^2+^ equivalent ratios ranging from 2.74 to 3.93 (Figure 6c), indicating that Ca^2+^ contributions exceeded those of Mg^2+^. In the context of the Nenjiang Formation, which consists of siltstone with calcareous cement and feldspar-bearing clasts, this ratio is consistent with combined dissolution of calcite cement (which supplies Ca^2+^ without Mg^2+^) and weathering of Ca-bearing silicate minerals (e.g., plagioclase). The very low SO_4_^2−^ concentrations (mean: 0.32 meq/L) further exclude evaporite dissolution as a meaningful source. In the (Ca^2+^ + Mg^2+^) versus (HCO_3_^−^ + SO_4_^2−^) diagram (Figure 6d), all samples plotted below the 1:1 equivalence line, indicating (Ca^2+^ + Mg^2+^) < (HCO_3_^−^ + SO_4_^2−^), which confirms that HCO_3_^−^ was derived from both carbonate and silicate weathering.

The cation exchange adsorption process in groundwater was evaluated using the Chloro-Alkaline Index (CAI-I and CAI-II) methods (Equations (10) and (11)) [52].

(10) $$\mathrm{CAI}\text{-}I=\frac{{\mathrm{Cl}}^{-}-K^{+}+{\mathrm{Na}}^{+}}{{\mathrm{Cl}}^{-}}$$

(11) $$\mathrm{CAI}\text{-}\mathrm{II}=\frac{{\mathrm{Cl}}^{-}-K^{+}+{\mathrm{Na}}^{+}}{{\mathrm{SO}}_{4}^{2-}+{\mathrm{HCO}}_{3}^{-}}$$

**Figure 7:**
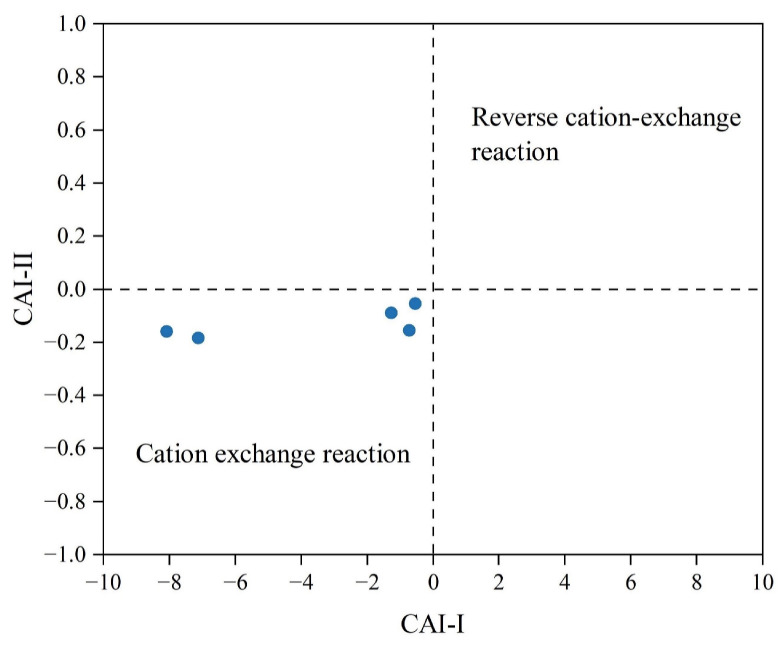
Chloro-alkaline indices (CAI-I and CAI-II) of groundwater samples. All values are negative, indicating reverse cation exchange (Ca^2+^ and Mg^2+^ in groundwater replace Na^+^ on the aquifer matrix, releasing Na^+^ into solution). Blue dots represent groundwater samples.

If Ca^2+^ and Mg^2+^ in the water replace Na^+^ and K^+^ from the aquifer medium, the index values are negative; conversely, they are positive. The larger the absolute values of CAI-I and CAI-II, the stronger the cation exchange adsorption effect [53]. As shown in Figure 7, the CAI-I and CAI-II values for the groundwater samples are all negative, indicating that cation exchange adsorption is one of the primary sources of Na^+^ in the groundwater of the study area. Specifically, Ca^2+^ and Mg^2+^ in the groundwater replace Na^+^ from the aquifer medium, leading to an increase in Na^+^ concentration in the groundwater.

### 4.2. Analysis of Factors Affecting Groundwater Quality

**Figure 8:**
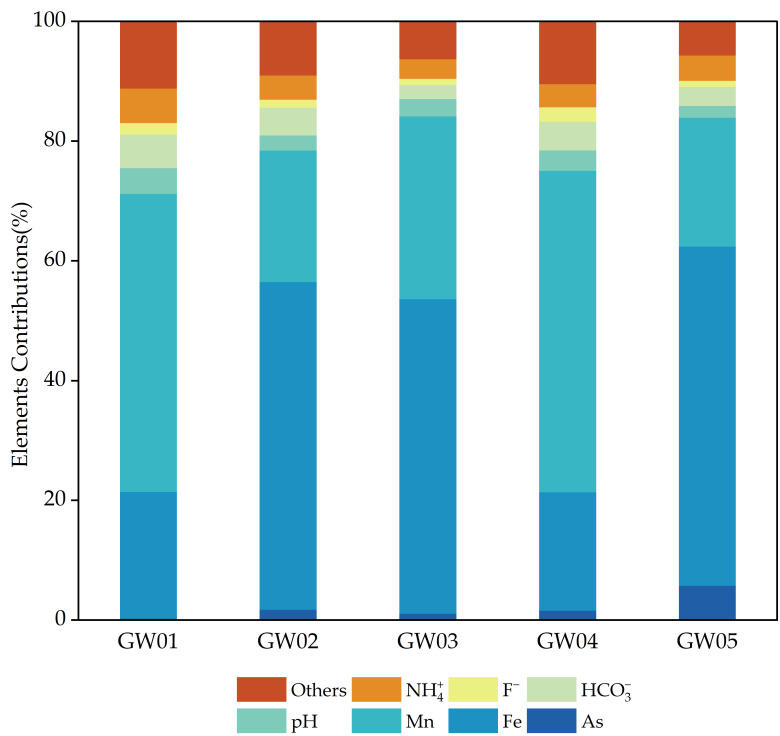
Contribution rates (%) of individual parameters to the WQI for each sampling site. Fe and Mn collectively account for 70.94–83.03% of the total WQI, identifying them as the primary factors degrading groundwater quality.

The overall groundwater quality in the study area was classified as moderate. As shown in Figure 8, the combined contribution of Fe and Mn to the WQI ranged from 71.60% to 83.60%, far exceeding that of other parameters, indicating that Fe and Mn were the primary factors degrading groundwater quality. The Songnen Plain strata are rich in Fe- and Mn-bearing minerals, and the high organic content in the soil, combined with reducing conditions at low altitudes, facilitates the dissolution of Fe and Mn into groundwater [54,55], resulting in elevated concentrations. This interpretation is consistent with previous studies on the Songnen Plain, which have consistently identified naturally elevated Fe and Mn as the dominant factors exceeding drinking water standards, attributable to the dissolution of Fe- and Mn-bearing minerals in the Cretaceous aquifer [13,16,56]. Similar findings have been reported in other regions with comparable geological settings, where Fe and Mn exceedances in deep groundwater were linked to natural weathering and dissolution of source minerals within sedimentary aquifers [57,58,59]. In summary, the elevated Fe and Mn concentrations in the deep groundwater of the study area are primarily controlled by natural geological factors rather than anthropogenic contamination.

### 4.3. Health Risk Prioritization and Vulnerable Populations

Non-carcinogenic and carcinogenic risks were evaluated for children, females, and males through drinking water ingestion and dermal contact pathways using the HHRA model. As shown in Figure 3, the non-carcinogenic risk via the drinking water pathway was higher than that via the dermal exposure pathway, and the risks for both pathways consistently decreased in the order: children > females > males. The contributions of individual substances to the non-carcinogenic risk were consistent across the three population groups for both exposu1re pathways, ranked as: As > F^−^ > Mn > Fe > NO_2_^−^ > Zn.

The hazard index (HI) values decreased in the order of children (0.988) > females (0.701) > males (0.534) (Figure 3), indicating that children were subject to the highest non-carcinogenic risk. All HI values were below the USEPA threshold of 1.0, suggesting that the overall non-carcinogenic risks were acceptable. Nevertheless, it is noteworthy that the HI for children (0.988) approached the threshold, with As and F^−^ as the dominant risk contributors. Moreover, although Fe and Mn ranked lower in the HQ-based prioritization due to their higher reference doses, their concentrations in the groundwater substantially exceeded the drinking water standards (Table 7). Epidemiological studies have confirmed that long-term exposure to elevated manganese levels is associated with neurological impairment, potentially increasing the risk of neurodegenerative diseases and adversely affecting children’s neurodevelopment [60,61]. These findings highlight the need for appropriate Fe and Mn removal measures before the groundwater is used for drinking purposes, particularly for vulnerable populations such as children.

### 4.4. Implications for Ecological Sustainability in Rural Areas

The findings of this study offer a preliminary scientific basis for promoting ecological sustainability in Changfa Town and the broader rural regions of the Songnen Plain. The moderate groundwater quality, dominated by naturally elevated Fe and Mn concentrations, highlights the need for targeted water quality management in rural areas where groundwater is the primary drinking water source. Although the health risks associated with groundwater consumption were below the USEPA thresholds, the elevated Fe and Mn concentrations can compromise water usability for domestic and agricultural purposes and may affect ecosystem health over the long term if left unmanaged. Aeration biofilters have been shown to be a sustainable, cost-effective, and energy-efficient intervention for Fe and Mn removal [62,63], which could be particularly suitable for rural households with limited infrastructure.

### 4.5. Limitations and Future Perspectives

Several limitations of this study should be acknowledged. First, although the five samples exhibited spatial variability at the township scale, the small sample size from a single township constrains the statistical power of the analysis and limits the representativeness of the results for the broader Songnen Plain. Second, the weights assigned to WQI parameters were derived from expert judgment and previous studies [22,23,24,26], which inevitably involve a degree of subjectivity. Different weighting schemes may yield slightly different WQI classifications, although the relative ranking of water quality among sampling sites is expected to remain consistent. Third, the HHRA model evaluates individual substances independently and does not account for cumulative or synergistic effects of multiple contaminants, which may underestimate the actual health risks.

Future research should expand the sampling network to include a greater number of wells across diverse geological formations and land use types, and incorporate multi-seasonal campaigns to capture temporal variability. Long-term monitoring of key parameters, particularly Fe and Mn, is needed to evaluate temporal trends and the effectiveness of remediation measures.

## 5. Conclusions

The groundwater in the study area was weakly alkaline (mean pH = 7.11), with mean TH and TDS of 103.88 mg·L^−1^ and 283.96 mg·L^−1^, respectively. The major ions followed the order of HCO_3_^−^ > Cl^−^ > SO_4_^2−^ for anions and Ca^2+^ > Na^+^ > Mg^2+^ for cations, classifying the hydrochemical type as HCO_3_-Ca type. The groundwater chemistry was primarily controlled by the weathering and dissolution of silicate and carbonate minerals, with cation exchange serving as a secondary process modifying the hydrochemical evolution.

The WQI ranged from 84.78 to 192.82 (mean = 132.68), indicating moderate overall groundwater quality. Naturally elevated concentrations of Fe and Mn were the primary factors degrading water quality. Although both the non-carcinogenic risk (HI) and carcinogenic risk (TCR) were below the USEPA thresholds for all population groups, the HI for children (0.988) approached the threshold, warranting continued attention to As and F^−^ as the dominant risk contributors.

As this study was based on a limited number of samples from a single season, the findings should be considered preliminary. Future research should expand the sampling network, incorporate multi-seasonal monitoring, and apply isotopic tracers to strengthen source identification and validate these baseline results.

## Figures and Tables

**Table 1:** Information of groundwater sampling wells in Changfa Town, Hailun City.

Sample Number	Well Depth (m)	Elevation (m)	Groundwater Type	Aquifer Type
GW01	120	209	Confined	Pore-crack aquifer
GW02	114	220	Confined	Pore-crack aquifer
GW03	117	217	Confined	Pore-crack aquifer
GW04	170	202	Confined	Pore-crack aquifer
GW05	183	201	Confined	Pore-crack aquifer

**Table 2:** Field and laboratory analytical methods and detection limits.

Tested Parameters	Analytical Methods	Detection Limit	Unit
pH	Potentiometric analysis method	0.1	-
TDS	Gravimetric method	4	mg·L^−1^
TH	Titrimetric method	1	mg·L^−1^
Na^+^	ICP-OES	0.03	mg·L^−1^
K^+^	ICP-OES	0.07	mg·L^−1^
Mg^2+^	ICP-OES	0.02	mg·L^−1^
Ca^2+^	ICP-OES	0.02	mg·L^−1^
HCO_3_^−^	Titrimetric method	2	mg·L^−1^
Cl^−^	Ion chromatography	0.06	mg·L^−1^
SO_4_^2−^	Ion chromatography	0.1	mg·L^−1^
F^−^	Ion chromatography	0.03	mg·L^−1^
NH_4_^+^	Ion chromatography	0.06	mg·L^−1^
NO_2_^−^	Ultraviolet-visible spectrophotometry	0.0002	mg·L^−1^
NO_3_^−^	Ion chromatography	0.02	mg·L^−1^
As	AFS	0.15	μg·L^−1^
Ba	ICP-OES	0.01	mg·L^−1^
Cr	ICP-OES	0.03	mg·L^−1^
Cu	ICP-OES	0.04	mg·L^−1^
Fe	ICP-OES	0.01	mg·L^−1^
Mn	ICP-OES	0.01	mg·L^−1^
Ni	ICP-OES	0.007	mg·L^−1^
Se	AFS	0.168	μg·L^−1^
Zn	ICP-OES	0.009	mg·L^−1^
CN^−^	Flow injection online distillation	2.0	μg·L^−1^
Volatile Phenols	Flow injection online distillation	0.002	mg·L^−1^

**Table 4:** Table of exposure dose parameter.

Parameter	Parameter Meaning	Units	Children	Males	Females
IR	Ingestion rate	L·d^−1^	1.14 ^a^	1.70 ^b^	1.70 ^b^
BW	Body weigh	kg	23.80 ^a^	65 ^c^	50 ^c^
ET	Bath time	h·d^−1^	0.3 ^c^	0.2 ^c^	0.5 ^c^
ABS	Gastrointestinal absorption factor	dimensionless	0.5 ^c^	0.5 ^c^	0.5 ^c^
AT	Average time	d	Noncarcinogenic: *ED* × 365 ^a^ Carcinogenic: 28,294.8 ^c^	Noncarcinogenic: *ED* × 365 ^b^ Carcinogenic: 28,294.8 ^c^	Noncarcinogenic: *ED* × 365 ^b^ Carcinogenic: 28,294.8 ^c^
EV	Bathing frequency	dimensionless	1.5 ^c^	2 ^c^	1 ^c^
K_p_	Dermal permeability coefficient	cm·h^−1^	0.001 ^d^	0.001 ^d^	0.001 ^d^
CF	Unit conversion factor	L·cm^−3^	0.001 ^d^	0.001 ^d^	0.001 ^d^
ED	Exposure duration	a	6 ^c^	30 ^c^	30 ^c^
EF	Exposure frequency	d·a^−1^	365 ^a^	365 ^b^	365 ^b^
SA	Exposed skin area	cm^2^	1.2 × 10^4 a^	1.6 × 10^4 b^	1.5 × 10^4 b^

^a^ These data are from [29]; ^b^ These data are from [30]; ^c^ These data are from [31]; ^d^ These data are from [32].

**Table 5:** *Rfd* and *SF* of elements.

Elements	*Rfd* (mg·kg^−1^·d^−1^)	*SF* (mg·kg^−1^·d^−1^)	Reference
Mn	0.14	-	[33]
Fe	0.7	-	[33]
Zn	0.3	-	[34]
NO_2_^−^	0.1	-	[34]
F^−^	0.06	-	[35]
As	0.0003	1.5	[35]

**Table 6:** Analytical results of the five samples.

Parameters	Unit	GW01	GW02	GW03	GW04	GW05
pH	-	6.96	6.98	7.16	7.17	7.31
TDS	mg·L^−1^	235.00	353.00	162.80	366.00	303.00
TH	mg·L^−1^	130.10	146.10	79.00	78.10	86.10
Na^+^	mg·L^−1^	16.40	23.80	13.00	52.80	19.40
K^+^	mg·L^−1^	1.16	1.32	1.02	1.09	1.18
Mg^2+^	mg·L^−1^	7.97	17.50	6.04	8.02	11.9
Ca^2+^	mg·L^−1^	51.60	79.10	29.00	51.20	71.60
HCO_3_^−^	mg·L^−1^	251.20	352.80	161.50	281.10	322.90
Cl^−^	mg·L^−1^	2.90	24.70	2.58	48.00	13.70
SO_4_^2−^	mg·L^−1^	0.86	28.40	4.02	39.90	2.91
F^−^	mg·L^−1^	0.37	0.43	0.30	0.60	0.43
NH_4_^+^	mg·L^−1^	0.43	0.51	0.38	0.38	0.72
NO_2_^−^	mg·L^−1^	ND	0.014	0.0011	0.0076	0.038
NO_3_^−^	mg·L^−1^	ND	ND	ND	ND	ND
As	mg·L^−1^	0.0006	0.0047	0.0027	0.0033	0.0200
Ba	mg·L^−1^	0.11	0.27	0.09	0.08	0.19
Cr	mg·L^−1^	ND	ND	ND	ND	ND
Cu	mg·L^−1^	ND	ND	ND	ND	ND
Fe	mg·L^−1^	1.21	5.31	4.59	1.48	7.35
Mn	mg·L^−1^	0.95	0.71	0.89	1.34	0.93
Ni	mg·L^−1^	ND	ND	ND	ND	ND
Se	mg·L^−1^	ND	ND	ND	ND	ND
Zn	mg·L^−1^	0.037	0.020	0.013	0.015	0.015
CN^−^	mg·L^−1^	ND	ND	ND	ND	ND
Volatile Phenols	mg·L^−1^	ND	ND	ND	ND	ND

-, dimensionless; ND, not detected.

**Table 7:** Statistical analysis of groundwater hydrochemical parameters.

Parameters	Unit	Min.	Max.	Mean	SD	CV	SDWQ	WHO
pH	-	6.96	7.31	7.11	0.15	0.02	6.5–8.5	6.5–8.5
TDS	mg·L^−1^	162.80	366.00	283.96	85.05	0.30	1000	1000
TH	mg·L^−1^	78.10	146.10	103.88	31.90	0.31	450	500
Na^+^	mg·L^−1^	13.00	52.80	25.08	16.00	0.64	200	200
K^+^	mg·L^−1^	1.02	1.32	1.15	0.11	0.10	-	-
Mg^2+^	mg·L^−1^	6.04	17.50	10.29	4.56	0.44	-	30
Ca^2+^	mg·L^−1^	29.00	79.10	56.50	19.67	0.35	-	200
HCO_3_^−^	mg·L^−1^	161.50	352.80	273.90	73.87	0.27	-	120
Cl^−^	mg·L^−1^	2.58	48.00	18.38	18.90	1.03	250	250
SO_4_^2−^	mg·L^−1^	0.86	39.90	15.22	17.79	1.17	250	250
F^−^	mg·L^−1^	0.30	0.60	0.43	0.11	0.26	1.0	1.5
NH_4_^+^	mg·L^−1^	0.38	0.72	0.47	0.13	0.29	0.5	-
NO_2_^−^	mg·L^−1^	ND	0.038	0.012	0.016	1.28	-	3.0
NO_3_^−^	mg·L^−1^	ND	ND	0.01	-	-	20	50
As	mg·L^−1^	0.0006	0.02	0.0063	0.0078	1.24	0.01	0.01
Ba	mg·L^−1^	0.08	0.27	0.15	0.08	0.55	0.7	1.3
Cr	mg·L^−1^	ND	ND	0.015	-	-	0.05	0.05
Cu	mg·L^−1^	ND	ND	0.02	-	-	1.0	2.0
Fe	mg·L^−1^	1.21	7.35	3.99	2.62	0.66	0.3	-
Mn	mg·L^−1^	0.71	1.34	0.96	0.23	0.24	0.1	-
Ni	mg·L^−1^	ND	ND	0.0035	-	-	0.02	0.07
Se	mg·L^−1^	ND	ND	0.00008	-	-	0.01	0.04
Zn	mg·L^−1^	0.013	0.037	0.02	0.01	0.49	1.0	-
CN^−^	mg·L^−1^	ND	ND	0.001	-	-	0.05	-
Volatile Phenols	mg·L^−1^	ND	ND	0.001	-	-	0.002	-

CV, the coefficient of variation; SD, standard deviation; -, dimensionless; ND, not detected; SDWQ, [26]; WHO, [27].

**Table 8:** Results of carcinogenic risk assessment.

Risk Type	Parameters	Children	Males	Females
CR_ing_	As	1.75 × 10^−5^	4.78 × 10^−5^	6.21 × 10^−5^
CR_derm_	As	1.66 × 10^−7^	3.60 × 10^−7^	5.48 × 10^−7^
TCR	Total CR	1.77 × 10^−5^	4.81 × 10^−5^	6.27 × 10^−5^

## Data Availability

The original contributions presented in this study are included in the article. Further inquiries can be directed to the corresponding author.
